# Immunophenotypic properties association of CLL and ALL patient cells by flow cytometry analysis

**DOI:** 10.1016/j.jtumed.2024.09.008

**Published:** 2024-09-24

**Authors:** Khder H. Rasul, Mohammed A. Wsoo, Dlshad H. Hassan, Shler Kh. Hamadamin, Zainab J. Qadr

**Affiliations:** a Department of Biology, College of Science, Salahaddin University-Erbil, Erbil, Kurdistan Region, Iraq; b Medical Analysis Department, Tishk International University, Erbil, Iraq; c Medical Laboratory Science, College of Science, University of Raparin, Ranya, Sulaymaniyah, Iraq; d Biology Department, Faculty of Science, Soran University, Soran, Erbil, Iraq

**Keywords:** ابيضاض الدم الليمفاوي المزمن, ابيضاض الدم الليمفاوي الحاد, الارتباط, الانحدار, التدفق الخلوي, مستضد سي دي 19, Acute lymphocytic leukemia, Chronic lymphocytic leukemia, Cluster of differentiation 19, Correlation, Flow cytometry, Regression

## Abstract

Chronic lymphocytic leukemia (CLL) and acute lymphoblastic leukemia (ALL) are blood cancers that affect lymphocytes and can be diagnosed by flow cytometry. Flow cytometry is a laboratory technique that analyzes cell properties, including cell surface markers such as cluster of differentiation 19 (CD19).

**Objective:**

The main objective of this study was to explore the correlation of the number of CD19-positive cells with other CD antigens in patients with CLL and ALL.

**Methods:**

After receiving ethical approval (Approval No. 5S/401), blood was collected from participants who had been diagnosed by a physician. Then the collected blood was prepared for flow cytometry analysis according to the protocol by staining with fluorescent antibodies.

**Results:**

The results of the current study showed that sex and different age groups had no statistical influence on the number of CD19-positive cells in the patients evaluated. The generated models did not reveal an association with the number of CD19-positive cells in patients with CLL and ALL. In patients with CLL, the number of cells expressing CD5, CD20, CD23, and CD200 was significantly and positively related with the number of CD19-positive cells. In patients with ALL, the number of cells expressing CD79 and CD99 was significantly and positively correlated with the number of CD19-positive cells. This comparison study also found that in patients with CLL, the number of CD19-positive cells was significantly higher than the number of cells expressing CD20, CD23, and CD200. In patents with ALL, there was a significantly higher number of CD19-positive cells than CD34-positive and CD79-positive cells.

**Conclusion:**

In patients with CLL, there was a strong positive correlation between the number of CD19-positive cells and the number of cells expressing CD5, CD20, CD23, and CD200. Additionally, in patients with ALL, there was a positive correlation of CD79 and CD99 with the number of CD19-positive cells.

## الملخص

**أهداف البحث:**

ابيضاض الدم الليمفاوي المزمن وابيضاض الدم الليمفاوي الحاد هما سرطانات دم تؤثر على الخلايا الليمفاوية ويمكن تشخيصها عن طريق قياس التدفق الخلوي. قياس التدفق الخلوي هو تقنية مخبرية تحلل خصائص الخلية، بما في ذلك العلامات السطحية مثل سي دي 19. تهدف هذه الدراسة إلى استكشاف العلاقة بين عدد الخلايا الإيجابية لمستضد سي دي 19 والعديد من المستضدات التمايزية الأخرى في مرضى ابيضاض الدم الليمفاوي المزمن والحاد.

**طرق البحث:**

تم جمع عينات الدم من المشاركين المشخصين من قبل الأطباء بعد الحصول على الموافقة الأخلاقية. تم تحضير العينات لتحليل التدفق الخلوي وفقا للبروتوكول باستخدام الأجسام المضادة المتألقة. تم تحليل البيانات باستخدام برنامج "فلوجو 10" وإجراء التحليل الإحصائي باستخدام برنامج "جراف باد بريزم الإصدار 9.0."

**النتائج:**

لم يظهر تأثير إحصائي للجنس والفئات العمرية المختلفة على عدد الخلايا الإيجابية لمستضد سي دي 19 في المرضى المعنيين. أظهرت النتائج وجود ارتباط إيجابي كبير بين عدد الخلايا الإيجابية لمستضد سي دي 19 وعدد الخلايا الإيجابية لمستضدات سي دي 5 وسي دي 20 وسي دي 23 وسي دي 200 في مرضى ابيضاض الدم الليمفاوي المزمن. في مرضى ابيضاض الدم الليمفاوي الحاد، وجد ارتباط إيجابي كبير بين عدد الخلايا الإيجابية لمستضد سي دي 19 وعدد الخلايا الإيجابية لمستضدي سي دي 79 وسي دي 99.

**الاستنتاجات:**

هناك ارتباط إيجابي قوي بين عدد الخلايا الإيجابية لمستضد سي دي 19 ومستضدات سي دي 5 وسي دي 20 وسي دي 23 وسي دي 200 في مرضى ابيضاض الدم الليمفاوي المزمن. في خلايا مرضى ابيضاض الدم الليمفاوي الحاد، هناك ارتباط إيجابي بين عدد الخلايا الإيجابية لمستضد سي دي 19 ومستضدي سي دي 79 وسي دي 99. يمكن استخدام المستضدات المرتبطة كعلامات داعمة عند تشخيص مستضد سي دي 19 في ابيضاض الدم الليمفاوي المزمن والحاد.

## Introduction

Leukemia is a group of hematologic cancers distinguished by the proliferation of abnormal lymphoid cells in the bone marrow. Based on rate of progression, leukemia can be classified as chronic lymphocytic leukemia (CLL) or acute lymphoblastic leukemia (ALL), which affect the blood and bone marrow.^1^ The most prevalent form of adult leukemia is CLL. CLL is a lymphoproliferative condition characterized by the growth of monoclonal, mature B cells in the bone marrow, lymphoid tissues, and peripheral blood.^2^ By contrast, young children experience ALL more frequently than adults. ALL is an aggressive, rapidly progressing form of leukemia that demands immediate treatment compared to CLL, and is typically an allogeneic stem cell transplant.^3^

Thus, CLL and ALL are two different hematological malignancies that require accurate and efficient diagnostic protocols for effective treatment and management.^4^ The current diagnostic approaches include a combination of complete blood cell count, bone marrow examination, and immunophenotyping, which are important for the identification of disease subtypes and guiding therapeutic decisions. Manual microscopic evaluation of stained blood and bone marrow samples has been one of the traditional methods for the diagnosis of leukemia. However, these manual diagnostic methods can be time-consuming, prone to errors, and less accurate, especially when performed by fatigued or stressed professionals.^5^ The advancements in science and technology have led to the development of different diagnostic methods, including imaging, immunophenotypic, and molecular diagnostics, that aim to improve the accuracy and efficiency of leukemia detection.^6^ Flow cytometry, as an example, is a powerful laser-based immunophenotyping technique that uses dyes to provide information on the subtypes of leukemia.^7^ Furthermore, flow cytometry is used to recognize cluster of differentiation (CD) markers in leukemic cells.^8^ CD markers are cell surface proteins that serve as identifying markers on the surface of CLL and ALL cells; for example, CD19 is a marker of B cells.^9^ CD19 is a transmembrane glycoprotein that serves as a biomarker for both healthy and cancerous B cells.^10^

In addition, CD19, CD22 (membrane and cytoplasm), and CD79a are the earliest markers for ALL of B-cell lineage.^10^ For CLL, a panel of CD19, CD5, CD20, and CD23 markers is commonly used for diagnosis.^11^ However, in borderline cases, additional markers are needed to make a diagnosis, and there may be interactions between CD markers in both CLL and ALL. In ambiguous immunophenotypes, indicators such as CD5, CD20, CD23, and CD200 for CLL and CD10, CD34, CD79, and CD99 for ALL, may help refine the diagnosis.

Therefore, the aim of the current research was to explore the relationship between the number of CD19-positive cells and the number of cells expressing other CD markers in CLL and ALL using flow cytometry. Identifying other CD antigens that may serve as diagnostic biomarkers for CLL and ALL will be useful for confirming the diagnosis.

## Materials and Methods

### Patients with CLL and ALL

The current study was conducted in 178 patients: 83 with CLL and 95 with ALL, who were diagnosed by physicians at Nana-Kaly Hospital (Erbil, Iraq). The study was approved by the Human Ethics Committee of the College of Science at Salahaddin University-Erbil (Erbil, Iraq) (Approval No. 5S/401).

### Cell staining for flow cytometry

Blood (1.5–2.5 mL) was drawn from patients through venipuncture and washed with phosphate-buffered saline (PBS) (10010023; Gibco, Waltham, MA, USA) by adding 3 mL PBS for each 100 μL blood, mixing by pipetting, and centrifuging for 5 min at 300×*g*. Then the supernatant removed, and the sediment was resuspended in 2 mL red blood cell lysis Versalyse™ solution (IM3648; ImmunoTech S.A.S., Marseille, France), vortexed for 3–5 s, and incubated for 10 min. After the incubation period, cells were centrifuged for 5 min at 300×*g*, and the sediment was washed twice with PBS as described. Then the cells were stained with different fluorophore-conjugated antibodies (BD Biosciences, San Jose, USA) for CLL (Table 1) and ALL (Table 2) for 20 min in the dark, followed by washing again with PBS. The cell pellet was resuspended in 0.5 mL PBS and run through a flow cytometer (BD FACSCanto™ II; BD Biosciences, Franklin Lakes, NJ, USA). BD FACSCanto™ clinical software was used to analyze the flow cytometry data obtained from the BD FACSCanto™ II system.

**Table 1:** List of flow cytometry antibodies used for the CLL panel.

Marker	Fluorophore	Cat No/Lot No	Vendor	City, State, Country
CD19	PE-Cy7	341113	BD	San Jose, CA, USA
CD5	PerCP-Cy5.5	341109	BD	San Jose, CA, USA
CD20	V450	642283	BD	San Jose, CA, USA
CD23	PE	341008	BD	San Jose, CA, USA
CD200	APC	655428	BD	San Jose, CA, USA

**Table 2:** List of flow cytometry antibodies used for the ALL panel.

Marker	Fluorophore	Cat No/Lot No	Vendor	City, State, Country
CD19	PE-Cy7	341113	BD	San Jose, CA, USA
CD10	APC	340922	BD	San Jose, CA, USA
CD34	PerCP-Cy5.5	347213	BD	San Jose, CA, USA
CD79	PE	340654	BD	San Jose, CA, USA
CD99	BV421	743040	BD	San Jose, CA, USA

### Statistical analyses

The flow cytometry data obtained were analyzed by FlowJo™ version 10 software (Ashland, OR, USA), and statistical analyses were performed with GraphPad Prism version 9.0 (San Diego, CA, USA). The Mann–Whitney test was used for comparisons between the two groups. One-way analysis of variance with the Kruskal–Wallis test was performed to evaluate the statistically significant differences among the three age groups. Multiple linear regression was used to identify associations between variables. Spearman's rank correlation was used to determine the relationship between the number of CD markers in CLL and ALL patients. P ≤ 0.05 was considered statistically significant.

## Results and discussion

### Demographic characteristics of patients with CLL and ALL

In the current study, the majority of patients involved were male (about 2-fold more than female). Of the 178 patients, 23 female and 60 male patients had CLL, whereas 38 female and 57 male patients had ALL. The age of CLL patients ranged between 35 and 90 years, and the age of ALL patients ranged between 1 and 68 years. All CLL patients were age 35 and above, with the majority between 52 and 75 years old. However, patients with ALL were younger than 68 years old, with the majority younger than 25 years old (Table 3). Leukemia can affect people of all ages and sexes, but some types of leukemia are more common in certain age groups. CLL, for example, is more common in older adults and ALL occurs most often in children. One study revealed that the median age at CLL diagnosis is 63 years old in China and 69 years old in the United States.^12^ The most prevalent pediatric cancer is ALL.^13^ Our findings are consistent with previous research in terms of the age distribution of patients with CLL and ALL.^14^^,^^15^

**Table 3:** Demographic properties of patients.

Properties	Groups	
	CLL n = 83	ALL n = 95
Sex (F/M)	23/60	38/57
Age (years)	64 (35–90)	5 (1–68)
0–25	0	4 (1–25)
26–50	45.5 (35–50)	34.5 (26–41)
51–75	65 (52–75)	58.5 (51–68)
76–100	81 (77–90)	0

Median (minimum–maximum) was used for age. Sex is not specified for age groups.

### Number of CD19-positive cells in patients with CLL and ALL

**Figure 1:**
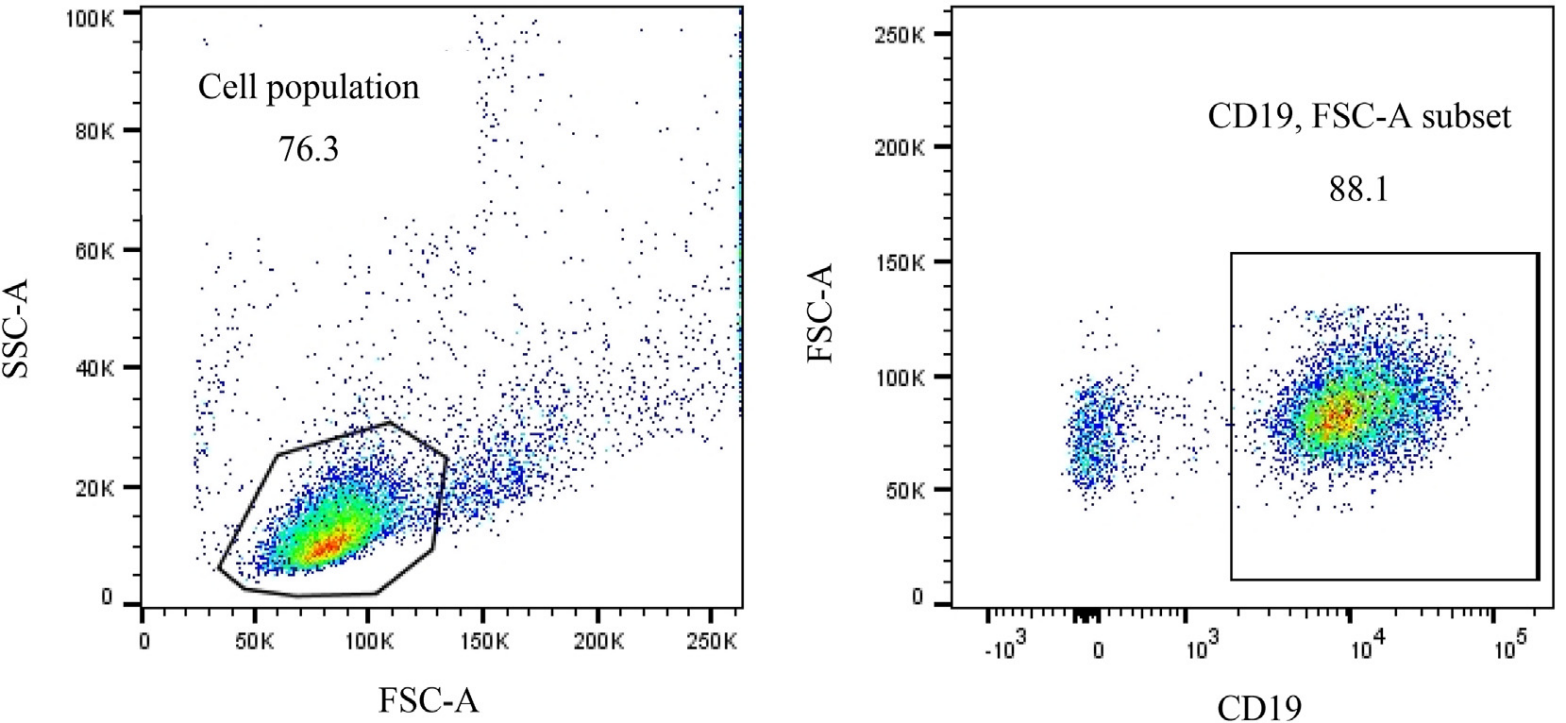
Flow cytometry dot plot images show the gating strategy; CD19 plots were gated on the gated cell population.

**Figure 2:**
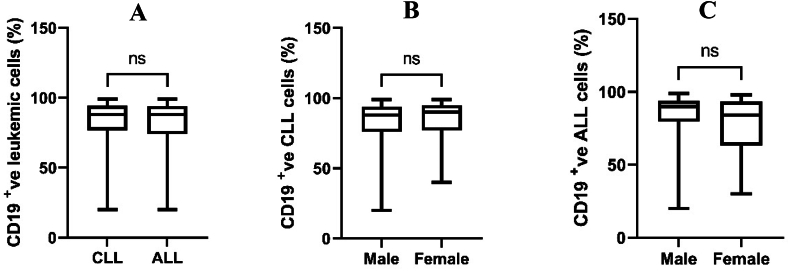
Comparison of CD19-positive leukemic cells. The Mann–Whitney U test was used for comparisons of (A) patients with CLL and ALL; (B) male and female patients with CLL; (C) male and female patients with ALL. ns, non-significant.

**Figure 3:**
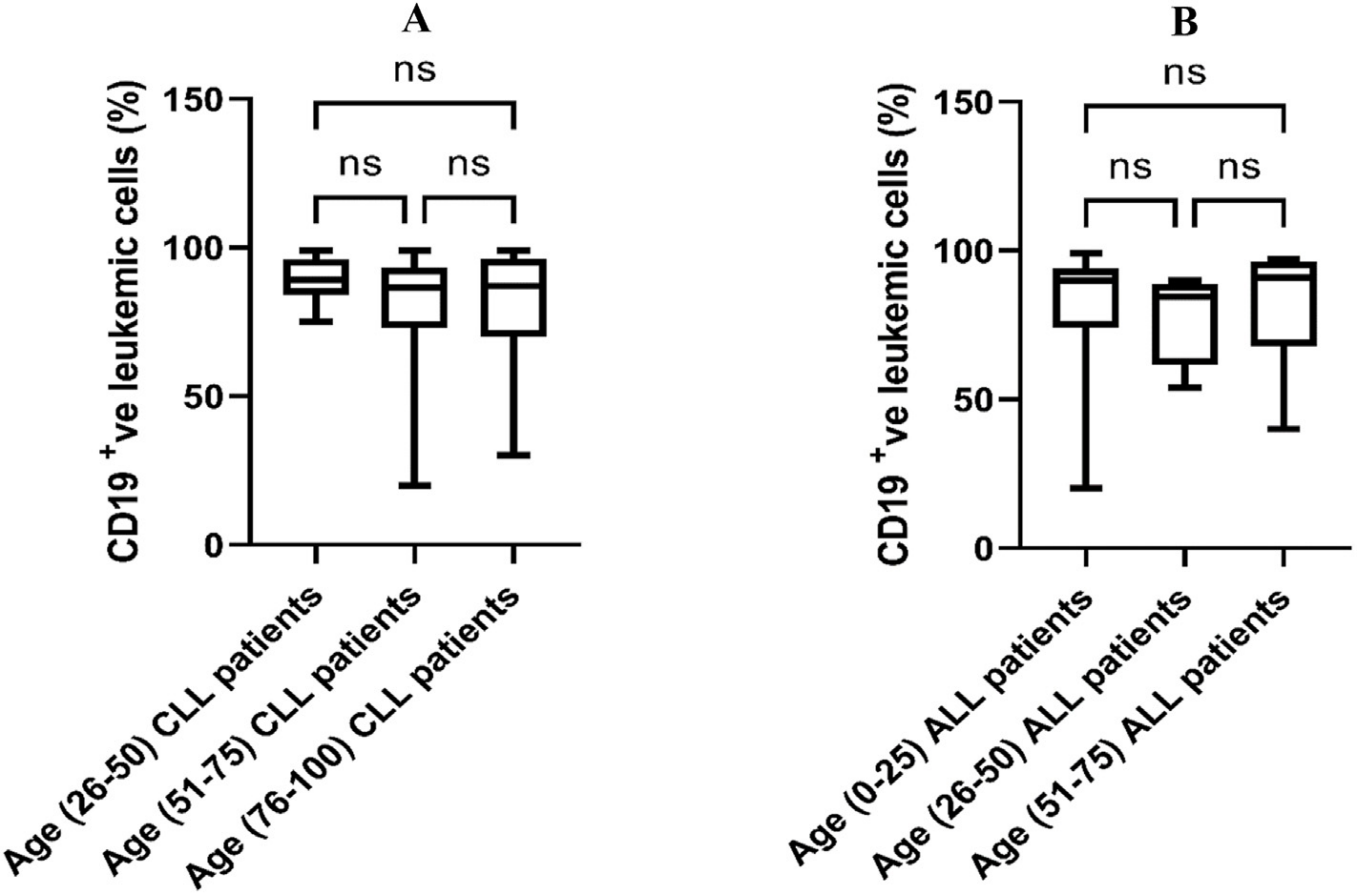
Comparison of CD19-positive leukemic cells. The Kruskal–Wallis test was used to compare (A) CLL among different age groups; (B) ALL among different age groups. ns, non-significant.

**Figure 4:**
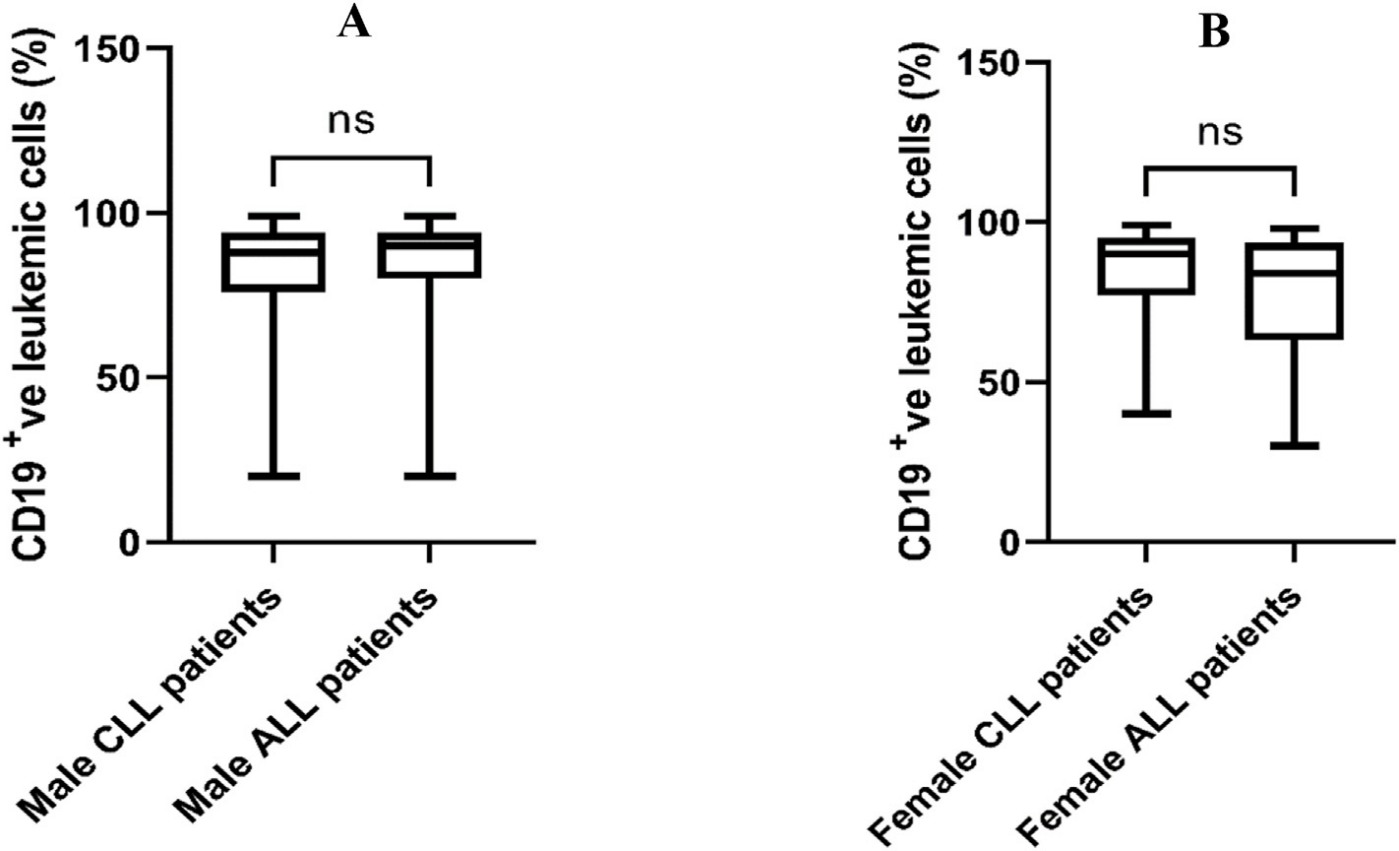
Comparison of CD19-positive leukemic cells of CLL and ALL. Mann–Whitney U test was used for comparisons of (A) male patients with CLL and ALL; (B) female patients with CLL and ALL. ns, non-significant.

**Figure 5:**
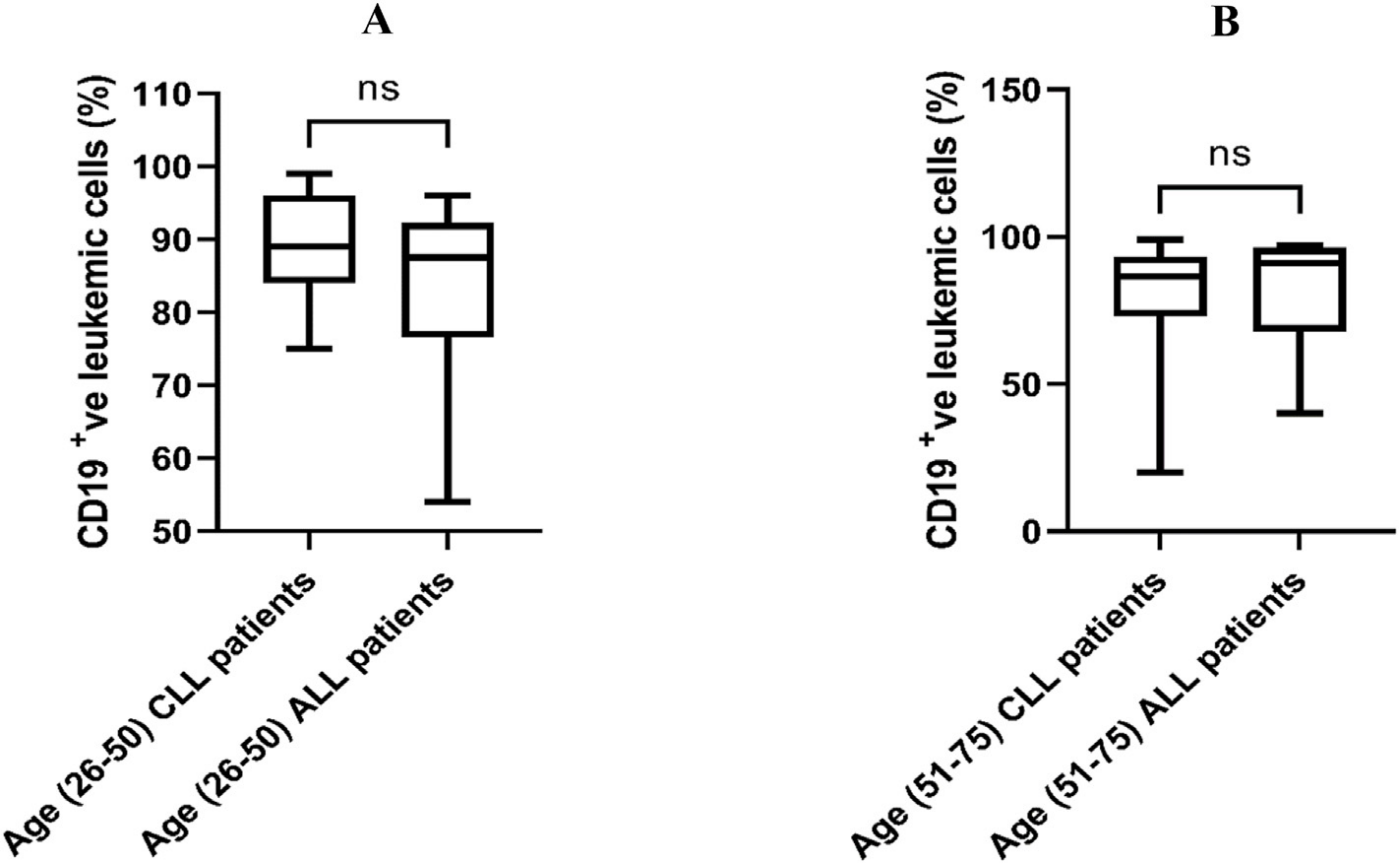
Comparison of CD19-positive leukemic cells between CLL and ALL. Mann–Whitney U test was used for comparisons between (A) CLL and ALL in the 26- to 50-year old age group; (B) CLL and ALL in the 51- to 75-year old age group. ns, non-significant.

The transmembrane glycoprotein CD19 strategy gating is displayed for the obtained flow cytometry data (Figure 1). Other CD plots were gated on the same gated cell population. In patients with both CLL and ALL, flow cytometry data showed that the number of CD19-positive cells was not significantly different between men and women (Figure 2), and also did not significantly differ among age groups (Figure 3). In addition, the number of CD19-positive cells in both male and female patients didn't reveal a significant difference between CLL and ALL patients (Figure 4). Moreover, the same age groups didn't discover the significant difference of the number of CD19-positive cells between CLL and ALL patients (Figure 5). The quantitative differences in CD19-positive cells between CLL and ALL are inherent to their pathophysiology and developmental stages. In the B-lymphocyte lineage, CD19 is the most widely expressed protein, similar to normal cells. CD19 expression increases at the time of B-lineage commitment during hematopoietic stem cell differentiation. It is then sustained through B-cell development and ultimately downregulated during terminal differentiation into plasma cells. In CLL and ALL, CD19 expression is commonly found on leukemic B cells.^16^^,^^17^ It is worth investigating the quantitative differences in CD19-positive cells between patients with CLL and ALL, but specific findings can depend on factors such as the disease stage and patient characteristics. However, overall evaluations of CD19-positive cells in CLL^18^ and ALL^19^ have not shown sex-specific differences in CD19 expression.

The rate of mutation increases with age, and leukemic transformation is clearly caused by particular genetic changes that prevent clonal B cells from undergoing apoptosis.^20^ The relationship between age and CD19-positive cells in patients with CLL is an important research topic in the field of oncology.^21^ Previous findings have shown that age does not affect the expression level of CD19 in ALL.^22^

### Comparison of the number CD19-positive cells with other CD-positive cells in patients with CLL and ALL

**Figure 6:**
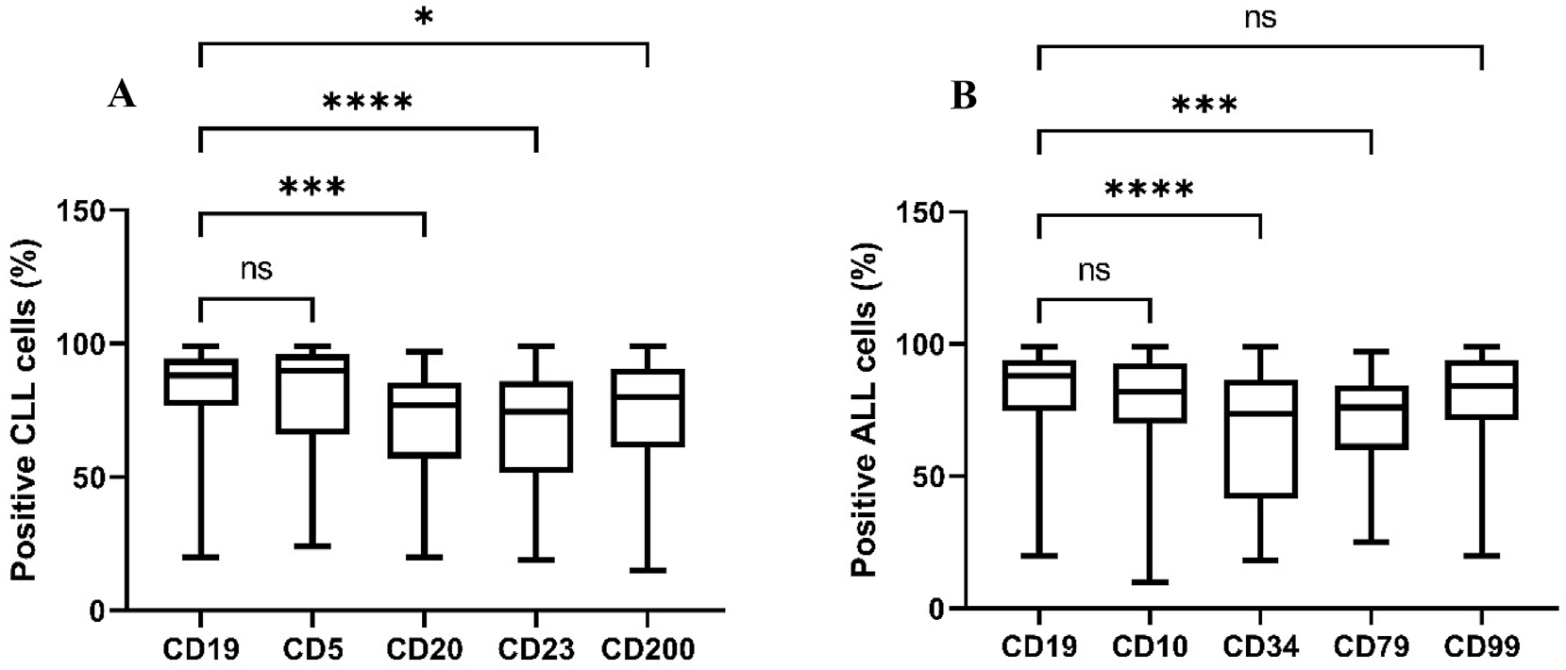
Comparison of CD-positive leukemic cells with CD19. The Kruskal–Wallis test was used for comparisons of (A) CD19 with CD5, CD20, CD23, and CD200 in patients with CLL; (B) CD19 with CD10, CD34, CD79, and CD99 in patients with ALL. ns, non-significant; ∗P < 0.05; ∗∗∗P < 0.001; ∗∗∗∗P < 0.0001.

In patients with CLL, flow cytometry data revealed that the number of CD19-positive cells was significantly higher than that of CD20- (P < 0.001), CD23- (P < 0.0001), and CD200- (P < 0.05) positive cells (Figure 6A). CD20 is expressed on the surface of B cells, from the pre-B cell stage until they differentiate into plasma cells. CD23 is expressed on the surface of B cells, activated macrophages, and some other cell types. CD200 is expressed on a variety of cells, including B cells, T cells, dendritic cells, and endothelial cells. CD200 plays a role in immune regulation by delivering inhibitory signals through its receptor, CD200R, which is expressed on myeloid cells and some lymphoid cells. In terms of patients with ALL, statistical analyses of the flow cytometry data showed a significantly higher number of CD19-positive cells compared with CD34- (P < 0.0001) and CD79- (P < 0.001) positive cells (Figure 6B). CD34 is one of the best known biomarkers of hematopoietic stem cell progenitors, and it is also expressed on a variety of cancer stem cells. CD34 has a variety of cellular functions such as the ability to enhance proliferation and inhibit cell differentiation, enhance lymphocyte adhesion, and play a role in cell morphogenesis.^23^ CD79 is composed of CD79a and CD79b components expressed almost exclusively on B cells and B-cell neoplasms.^24^ CD79a and CD79b are essential components of the B-cell receptor that are indispensable for its functionality, signal initiation, and signal transduction.^25^ Our results provide an understanding of the relative expression levels of other markers such as CD20, CD23, CD200, CD34, and CD79 within the context of the mentioned diseases, which could be beneficial for further research or clinical applications. CLL cells co-express the T-cell antigen CD5 and the B-cell surface antigens CD19, CD20, and CD23.^26^ In addition, CLL is one of the most common types of leukemia in adults, and is usually associated with high levels of CD5, CD19, and CD23, as well as human leukocyte antigen-DR (HLA-DR) expression.^27^^,^^28^ Furthermore, immunophenotyping of circulating B lymphocytes, which identify a clonal B-cell population carrying the CD5 antigen, as well as typical B-cell markers,^20^ has shown that CD200 can also be effective in activating B-cell CLL via different pathways.^29^ Regarding ALL, flow cytometry analyses have shown the expression of CD19, CD22, CD10, CD34, and HLA-DR markers by leukemic blasts.^30^ CD34 and CD10 expression is also frequent in ALL.^31^ CD79-positive cells are more common in patients with ALL.^32^ Therefore, the expression of these markers is crucial for the accurate diagnosis of patients with ALL.

### Multiple linear regression analysis of the relationship between several variables and the number of CD19-positive cells in patients with CLL

Multiple linear regression models were used to determine if there was an association between variables (sex, age, CD5, CD20, CD23 and CD200) and the number of CD19-positive cells. Variables were added to the models and acted as predictors for the number of CD19-positive cells. Multiple linear regression analyses of sex, age, CD5, CD20, CD23, CD200, and CD19 showed that models 1 (sex), 2 (sex and age), and 3 (sex, age, CD5, CD20, CD23, and CD200) were not statistically associated with the number of CD19-positive cells in patients with CLL. However, among the independent variables in model 3, CD200 was strongly associated and acted as a predictor of an increase in the number of CD19-positive cells in patients with CLL (Table 4). The transmembrane protein CD200, which is expressed on different cell types, binds to receptors to give immunoregulatory signals. Signals sent through the CD200-receptor axis have a big effect on antitumor immunity. High levels of CD200 have been linked to a number of cancers, including CLL, as well as cancer stem cells.^33^ Age, total leukocyte count, absolute lymphocyte count, and high levels of CD5, CD19, and CD23 are all correlated with high CD200 expression (≥50%).^34^

**Table 4:** Multiple linear regression of sex, age, CD5, CD20, CD23, and CD200 to predict CD19-positive cells in patients with CLL.

Dependent Variable	Model	Independent Variable	t	P-Value Summary
CD19	Model 1	Sex	0.8779	ns
	Model 2	Sex Age	0.3654 1.269	ns ns
	Model 3	Sex Age CD5 CD20 CD23 CD200	0.3481 0.1570 0.1234 1.814 0.0206 3.741	ns ns ns ns ns ∗∗∗

Abbreviations: t, regression coefficient; ns, non-significant; ∗∗∗P < 0.001.

### Multiple linear regression analysis of the relationship between several variables and the number of CD19-positive cells in patients with ALL

Multiple linear regression models were created to investigate the association between variables (sex, age, CD10, CD34, CD79, and CD99) and the number of CD19-positive cells. Variables were introduced into the models to serve as predictors of the number of CD19-positive cells. Multiple linear regression analysis of sex, age, CD10, CD34, CD79, CD99, and the number of CD19-positive cells in patients with ALL revealed that models 1 (sex), 2 (sex and age), and 3 (sex, age, CD10, CD34, CD79 and CD99) were not statistically associated with the number of CD19-positive cells (Table 5). CD19 is a B cell lineage-specific antigen that is expressed on malignant B cells in healthy cells and those from patients with ALL. Total leucocyte count, peripheral blood blast percentage, and platelet count were all correlated with greater expression levels of CD34 and CD10.^31^ In addition, in B-cell ALL, the B-cell precursor marker CD19 was expressed in all patients, and CD10 was expressed in 86.7% of patients.^35^

**Table 5:** Multiple linear regression of sex, age, CD10, CD34, CD79, and CD99 to predict CD19-positive cells in patients with ALL.

Dependent Variable	Model	Independent Variable	t	P-Value Summary
CD19	Model 1	Sex	1.509	ns
	Model 2	Sex	1.320	ns
		Age	0.6201	ns
	Model 3	Sex	0.6644	ns
		Age	1.340	ns
		CD10	0.2315	ns
		CD34	0.6898	ns
		CD79	1.925	ns
		CD99	0.4904	ns

Abbreviations: t, regression coefficient; ns, non-significant.

### Correlation among CD19-positive cells and the number of cells expressing CD19, CD5, CD20, CD23, and CD200 in patients with CLL

Spearman's rank correlation was used to analyze the correlation among CD19-positive cells and the numbers of cells expressing CD19, CD5, CD20, CD23, and CD200 in patients with CLL. We found that CD5 (P < 0.0001), CD20 (P < 0.01), CD23 (P < 0.0001), and CD200 (P < 0.0001) were positively and significantly correlated with the number of CD19-positive cells (Table 6). Transmembrane CD19 has become an important marker in CLL, and progress in anti-CD19 CAR T-cell therapies has shown promise in treating CLL. CD5-positive CLL cells often demonstrate more aggressive clinical behavior and resistance to apoptosis, in accordance with the study by Herling et al.,^36^ which showed that CD5 expression contributes to clonal dynamics and resistance to therapies. Therefore, the correlation between the numbers of CD19- and CD5-positive cells may have implications for targeted therapies. Consequently, research is needed to determine whether CD19 and CD5 expression profiles can predict treatment responses and guide therapeutic decision-making.^37^ Patients with trisomy 12 CLL exhibited substantial leukemic cell CD20 expression and a high response rate to rituximab-based therapy, a well-known B cell-associated antigen.^38^ CLL cells express the transmembrane receptor CD23 in addition to B-cell markers.^39^ Regarding CD200, one study showed that CLL samples were strongly positive for CD200.^40^

**Table 6:** Correlation analysis among independent markers of patients with CLL.

Marker (Y-axis)	Marker (X-axis)	r	P-Value Summary
CD19	CD5 CD20 CD23 CD200	0.4341 0.3957 0.5618 0.6490	∗∗∗∗ ∗∗ ∗∗∗∗ ∗∗∗∗

Abbreviations: r, correlation coefficient; ∗∗P < 0.01; ∗∗∗∗P < 0.0001.

### Correlation between the number of CD19-positive cells and cells expressing CD19, CD10, CD34, CD79 and CD99 in patients with ALL

As shown in Table 7, Spearman's rank correlation was used to analyze the correlation between the number of CD19-positive cells and the number of cells expressing CD19, CD10, CD34, CD79, and CD99 in patents with ALL. The results showed a significant positive correlation between the number of cells expressing CD79 (P < 0.05) and CD99 (P < 0.01) and CD19-positive cells. CD10 is a cell surface peptidase expressed in the early stages of B-cell differentiation. Flow cytometry showed that patients with ALL displayed a comparable blast phenotype with varying CD20 expression and co-expression of CD19, CD10, and TdT.^41^ A considerable proportion of patients with ALL do not have CD34 expressed on the surface of their leukemic cells.^42^ However, Cuéllar-Mendoza et al.^43^ and Enein et al.^44^ showed that patients with ALL expressed CD79 and CD99, respectively.

**Table 7:** Correlation analysis among independent markers of patients with ALL.

Marker (Y-axis)	Marker (X-axis)	r	P-Value Summary
CD19	CD10 CD34 CD79 CD99	0.1130 0.2447 0.2548 0.3006	ns ns ∗ ∗∗

Abbreviations: r, correlation coefficient; ns, non-significant; ∗P < 0.05; ∗∗P < 0.01.

## Conclusion

The sex and age of patients with CLL and ALL had no effects on the number of CD19-positive cells. In patients with CLL, CD5, CD20, CD23, and CD200 was strongly positively correlated with the number of CD19-positive cells. Also, in patients with ALL, CD79 and CD99 were positively correlated with the number of CD19-positive cells. In addition to CD19, other CDs that are expressed in CLL and ALL may serve as supportive markers for the diagnosis of patients with CLL and ALL.

## Source of funding

This research did not receive any specific grant from funding agencies in the public, commercial, or not-for-profit sectors.

## Conflict of interest

The authors have no conflict of interest to declare.

## Ethical approval

The present study was approved (approval no. 5S/401) by the Human Research Ethics Committee of Affiliated Salahaddin University (Erbil, Iraq).

## Authors contributions

KHR analyzed the data and wrote the manuscript. MAW and DHH are involved in data interpretation and manuscript editing. SKH and ZJQ collected data. All authors have read and agreed to the published version of the manuscript. All authors have critically reviewed and approved the final draft and are responsible for the content and similarity index of the manuscript.
